# Predicting the cross-continental spread of the cassava brown streak disease epidemic in sub-Saharan Africa

**DOI:** 10.1038/s41598-026-59438-2

**Published:** 2026-07-02

**Authors:** David Godding, Richard O. J. H. Stutt, Merveille Koissi Savi, Corneille Ahanhanzo, Fidèle Tiendrebeogo, Oumar Doungous, Monde Godefroid, Zeyimo Bakelana, Jacques François Mavoungou, Allen Oppong, Elijah Ateka, Willard Mbewe, Nurbibi Cossa, Chukwuemeka Nkere, Ibrahim Umar Mohammed, Marie Claire Kanyange, Alusaine Edward Samura, Adjata Djodji, Titus Alicai, Patrick Chiza Chikoti, Fred Tairo, Peter Sseruwagi, Joseph Ndunguru, Angela Obiageli Eni, Justin Simon Pita, Christopher A. Gilligan

**Affiliations:** 1 Epidemiology and Modelling Group, Department of Plant Sciences, University of Cambridge, Cambridge, CB2 3EA UK; 2 Central and West African Virus Epidemiology (WAVE), Scientific and Innovation Hub of Bingerville, Félix Houphouët- Boigny University (UFHB), Abidjan, 22 BP 582 Côte d’Ivoire; 3 Central and West African Virus Epidemiology (WAVE), Biotechnology Laboratory, Ekona Regional Research Centre, Institute of Agricultural Research for Development, PMB 25, Buea, Cameroon; 4 Institut de Recherches Agronomiques et Forestières (IRAF), B.P : 2246, Libreville, Gabon; 5 CSIR Crops Research Institute, P. O. Box 3785, Kumasi, Ghana; 6 Department of Horticulture, Jomo Kenyatta University of Agriculture and Technology, Nairobi, Kenya; 7 Jaramogi Oginga Odinga University of Science and Technology, Bondo, Kenya; 8 Department of Biological Sciences, Malawi University of Science and Technology, P. O. Box 5196, Limbe, Malawi; 9 Mozambique Agricultural Research Institute, Maputo, 2698 Mozambique; 10 National Root Crops Research Institute (NRCRI) Umudike, Km 8 Ikot Ekpene Road, PMB 7006 Umuahia, Nigeria; 11 Abdullahi Fodio University of Science and Technology, Aliero (AFUSTA), Along Sokoto Road, Aliero, PMB 1144 Kebbi State Nigeria; 12 Rwanda Agriculture Board, PO Box 5016, Kigali, Rwanda; 13 Department of Crop Protection, School of Agriculture and Food Sciences, Njala University, Njala Campus, Njala, Sierra Leone; 14 National Crops Resources Research Institute, Namulonge, Kampala Uganda; 15 Zambia Agriculture Research Institute, Ministry of Agriculture, Chilanga, Zambia; 16 Mikocheni Agricultural Research Institute, P.O. Box 6226, Dar es Salaam, Tanzania

**Keywords:** Computational biology and bioinformatics, Diseases, Ecology, Ecology, Mathematics and computing

## Abstract

Cassava brown streak disease (CBSD) is a major threat to smallholder farmers in sub-Saharan Africa (SSA), where cassava is a staple crop. Caused by cassava brown streak ipomoviruses (CBSIs), CBSD has spread extensively since its detection in Uganda in 2004, raising concerns about ongoing spread through Southern and Central Africa and potential expansion to West Africa, home to the world’s largest cassava producer, Nigeria. Building on a stochastic epidemiological model that predicts CBSD spread at the scale of Uganda, we incorporate extensive field surveillance records to extend the model to all thirty-two major cassava-producing countries in SSA. We then deploy the model to address key strategic questions such as estimating the present day CBSD distribution and predicting rates of ongoing spread towards West Africa. We also evaluate the risk of direct introductions resulting from extreme cases of long-range movement of infected planting material by air, sea or land, which could trigger outbreaks far beyond normal dispersal limits. Our model predicts the likely arrival of CBSD in Nigeria via cross-continental spread within 25 years, and if directly introduced anywhere in West Africa, spreading to most West African nations within 10 years of introduction. The risks of ongoing and future CBSD spread highlighted in this study underscore the need for proactive phytosanitation measures, including clean seed programs, vector control, and quarantine policies to curb CBSD spread. Moreover, the model described in this study not only provides estimates for arrival times across SSA, but also lays the foundations for a continental-scale quantitative framework wherein both surveillance and management options can be explored and optimised.

**Supplementary Information:**

The online version contains supplementary material available at https://doi.org/10.1038/s41598-026-59438-2.

## Introduction

Emerging epidemics and pest infestations of agricultural crops are a major burden for smallholder farmers in sub-Saharan Africa (SSA), causing income loss and threatening food security^1^. Smallholder farmers often have limited access to resistant varieties and pesticides to mitigate the continually shifting burden of pests and pathogens, in contrast to industrialised agriculture^2^. Therefore, smallholder farmers are most vulnerable to the yield losses that novel pests and pathogens can cause. Current and recent threats to staple crops in SSA include a range of transboundary pests and pathogens that spread within and between countries. Some, such as desert locusts^3^, experience population explosions under favourable environmental conditions, leading to large swarms that disperse over long distances^4,5^. Fall armyworm also spread rapidly across national boundaries^6^ following the first introductions in West and Central Africa^7^. Other disease-causing pathogens, such as cassava mosaic virus and cassava brown streak virus are less explosive but capable of spreading over long distances by human-mediated and insect vector transmission^8–12^.

Slower spreading outbreaks allow time to prepare surveillance, early-warning, and mitigation strategies in disease-free regions prior to invasion. Predicting likely arrival times and spread within a new region is an essential component for forward planning. Here we focus on cassava brown streak disease (CBSD) and investigate the use of stochastic, epidemiological modelling to assess the risk to West and Central Africa from cross continental spread and the alternative of a direct introduction via movement of infected planting material by air, land or sea routes over extreme long distances beyond normal dispersal, bypassing intervening countries and giving less warning time to countries at risk.

Since its first detection in Uganda in 2004, an epidemic of cassava brown streak disease has spread over 1000 km from Uganda^11,13^ to Rwanda^14^, Burundi^13^, western Kenya^15^, the lake-zone of Tanzania^16^, central Democratic Republic of Congo^17^, and Zambia^18^. The CBSD epidemic is having a major impact on the consumable and marketable yields of cassava for smallholder farmers, which threatens food security and economic development^10^ and is continuing to spread, threatening production in West Africa, which includes the world’s largest producer, Nigeria^19^.

The CBSD epidemic is caused by single-stranded ipomoviruses, Cassava brown streak virus (CBSV) and Ugandan cassava brown streak virus (UCBSV), of the family *Potyviridae*^20^, henceforth referred to collectively as cassava brown streak ipomoviruses (CBSIs). Cassava is vegetatively propagated, with stem cuttings taken from plants in one season used as planting materials in the next season, rather than planting from seed. As a result, in the absence of management efforts, once cassava plants in a field become infected with CBSD, they remain infected. Currently, there are no cassava varieties that are fully resistant to CBSD in SSA^21^, and all West African varieties tested to date are susceptible to infection^22,23^. However, there are significant efforts to identify sources of resistance that are showing promise^24^. Until resistant varieties are widely available, management options at the larger scale involve phytosanitation programmes. These include clean seed programmes to disseminate uninfected cuttings and quarantine systems to reduce the movement of infected planting material^10^. At the field level, options include: vector control with pesticides^25^ to reduce transmission rates; roguing to remove infected plants; and selection of asymptomatic cuttings for propagation to bias the choice of cuttings away from those that are infected^10^. Each of these management options has different requirements in terms of investment and training, and varying probabilities of success according to the epidemiological and social context.

CBSIs are spread by an insect vector, *Bemisia tabaci*, and by human-mediated movement of infected cuttings. Vector activity operates primarily at a local scale tospread infection, with cutting movement introducing the pathogen to new areas. The majority of cuttings are sourced from farmers’ own fields, but it is not uncommon to source from within a 10 km radius from friends and relatives^26^. More rarely, farmers may source materials from further afield (i.e., greater than 50 km), which are likely from either traders or not-for-profit organisations such as NGOs^26^. Therefore, when assessing how CBSD can spread to new regions, two distinct scenarios emerge. The first scenario captures the cumulative effect of relatively local and continuous spread with a strong local bias and occasional medium-range dispersal up to approximately 50 km, resulting from the combined effect of vector activity and local cutting movements^12,26^. The second scenario captures the risk of direct introduction of infected cuttings to a previously disease-free region over a long distance by air, sea, or long-range land transport of cuttings. This could introduce CBSIs to West Africa at any time, without prior spread through neighbouring countries, or advance warning. Beyond private actors, NGOs have been known to move cassava cuttings by air^27^, further contributing to this risk. Although long-range direct introduction events are likely to be rare, even a single event could trigger a large-scale independent outbreak at an unpredictable time.

Sparse surveillance reports from Eastern, Southern and Central Africa obscure the true extent of the CBSD epidemic in SSA, particularly the risks facing Central and West African countries, where cassava is a critical staple crop for both rural and urban populations. While CBSIs have been recorded in the Democratic Republic of Congo (DRC)^17^ and Zambia^18^, to date, there are no epidemiological modelling studies to predict the future spread of CBSIs to guide policy makers across Central and West Africa in preparedness, monitoring, and management efforts. Godding et al.^12^ recently analysed the spread of CBSD in Uganda in order to estimate dispersal parameters at country-wide scales. The stochastic, spatiotemporal epidemic model incorporates estimates of the cassava crop density and connectivity of the landscape (the probability that local dispersal events reach new hosts) alongside the whitefly vector density to simulate pathogen spread. The model, which was resolved to 1 km^2^, successfully predicted rates and extents of spread of CBSD throughout Uganda during the period 2004-2017^12^, for which well-documented surveillance data were available^11^. In this paper, we extend and evaluate the model developed in Uganda to predict likely cross-continental spread rates for all thirty-two major cassava-producing countries of SSA. Specifically, we use the spatially extended model to address the following epidemiological questions:

- How accurately does the model predict the post-2004 regional spread of CBSD?
- How can real-world reports of CBSD spread be incorporated into the model to reduce uncertainty in the predictions?
- What would be the impact of the direct introduction of CBSI-infected planting material to disease-free regions of West Africa?
- What is the current spatial distribution of the CBSD epidemic?
- What are the predicted arrival times for CBSIs to currently disease-free countries across sub-Saharan Africa in the absence of long-range direct introduction to disease-free regions?
- How much uncertainty is there in predictions of cross-continental spread?

In answering some of these questions, we also introduce innovations to reduce uncertainty in predicting risk by selecting epidemiological trajectories from large ensembles of simulations that account for limited positive records of disease occurrence in a sparsely sampled heterogeneous landscape.

## Results

### Validating predictions of regional spread from 2004 to the present day

**Fig. 1:**
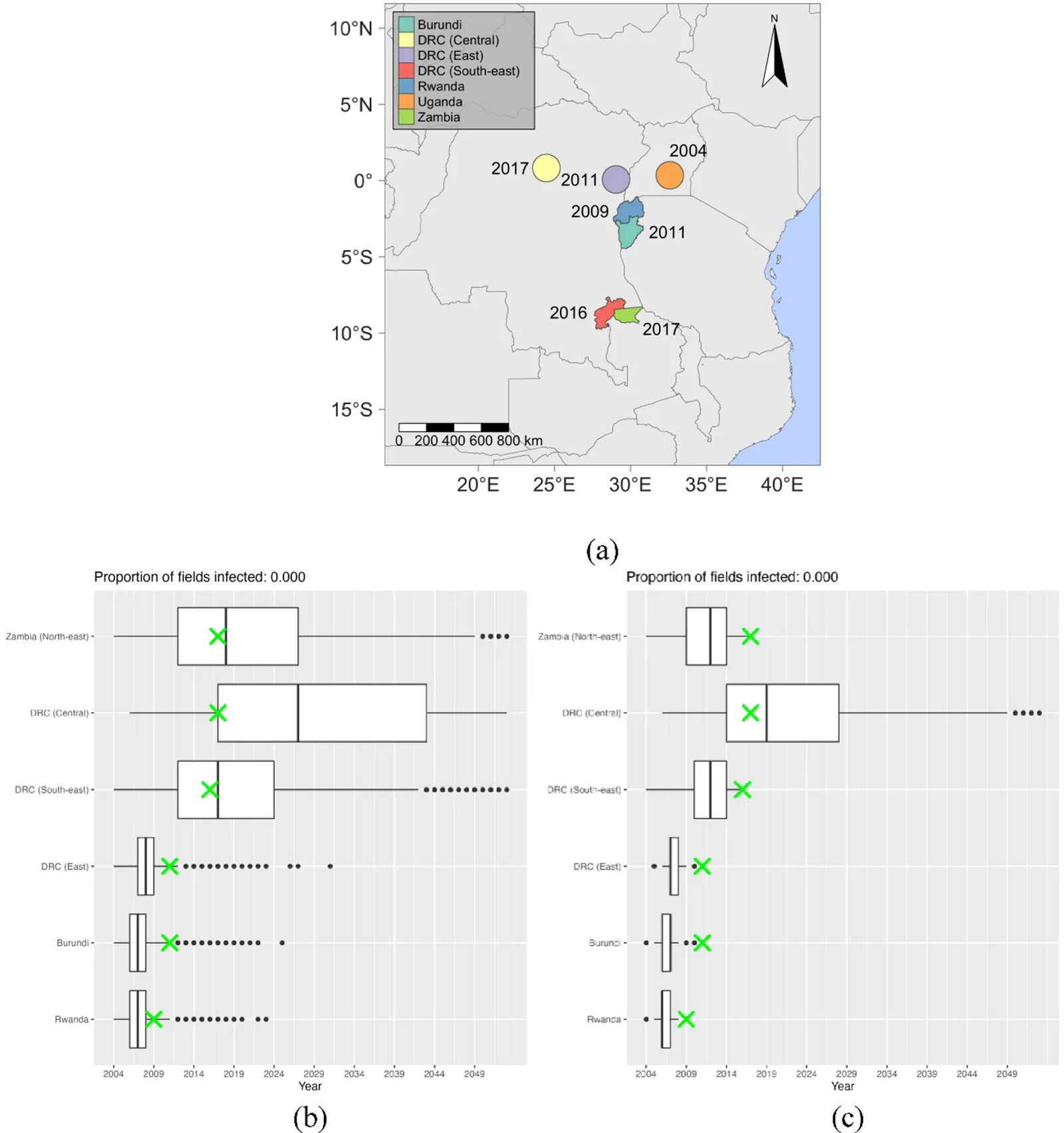
(**a**): Key observations of CBSD spread since the initial outbreak in Uganda in 2004. (**b**): Predicted arrival times in target regions compared with timing of real-world reports generated from all 9072 simulations initialised with infection only present at the sites of the first finding of CBSD in Uganda in 2004. Boxes indicate the median and the 25th and 75th percentiles; green crosses indicate year of real-world reporting. (**c**): Predicted arrival times in target regions compared with timing of real-world reports generated from a subset of 991 simulations out of the full set of 9072 simulations that corresponded to CBSD spread in five of the six regions (all except DRC (Central)) and surveillance data within Uganda.

Since the first observations of CBSD in Uganda in 2004, the epidemic has rapidly spread within and beyond Uganda. Previous studies have reported the strong correspondence of the model to surveillance data within Uganda during model validation^12^. Beyond Uganda, a limited number of reports document CBSD arrival in previously disease-free regions^11,13,14,17,18,28^(Fig. 1a).

From an ensemble of 9072 stochastic simulations for the spread of CBSI, beginning in 2004 within Uganda, we contrasted the predicted epidemic arrival times with the real-world reports of CBSD detection (Fig. 1b). Given that the model^12^ predicts the full state of the system, we would expect the model to predict CBSD arrival prior to or in the same year as the real-world reporting. The lag between actual arrival time of the pathogen and reporting should shorten as surveillance efforts increase. For five of the six CBSD regions, the model predictions correspond closely with the real-world reporting year. In the outlier case of Central DRC in which the report is comparatively early, the report still lies within the 25th percentile of simulations. Notably, the southeast DRC and Zambia reports in 2016 and 2017, respectively, are both approximately 1000 km from the original reports of the 2004 Ugandan outbreak. Nonetheless, the model predicts median arrival time within 1 year of the real-world report.

For forward predictions, we then incorporate additional real-world spread information to refine arrival time predictions. We generate a present-day (2025) infection status for all cassava hosts, including unsurveyed locations, to act as the initial conditions for forward simulations. To characterise the full current infection status, we ran a set of simulations from 2004 to the present day. We then retained only those simulations whose outputs matched present-day survey data and used those simulations as initial conditions for forward simulations (see Methods and Discussion for further information). Specifically, we filtered the 9,072 simulations to a subset of 991 simulations that matched verified reports of the disease within Uganda, Rwanda, Burundi, eastern DRC, southern DRC and Zambia, but did not apply this constraint to the central DRC report. Comparing Fig. 1b with Fig. 1c, incorporating real-world observations reduced the lag in median predicted arrival time for central DRC from 10 years to 2 years, demonstrating improved accuracy.

### Direct introduction of CBSI to disease-free regions in West Africa

In order to explore the impact of CBSI being introduced directly, for example by inadvertent import and planting of infected cassava to different regions in West Africa, in the absence of management efforts, we performed a simulated introduction of CBSI infected material at five independently selected cassava fields close to each of three major cities in West Africa: Lagos, Nigeria; Abidjan, Côte d’Ivoire; and Douala, Cameroon, each with significant connections to international trade. Crucially, such an introduction could occur at any time. The time of introduction is not fixed to mimic the initiation of the epidemic at a point within the decade following 2025 (see Methods for definition of field and details of infection seeding methodology).

**Fig. 2:**
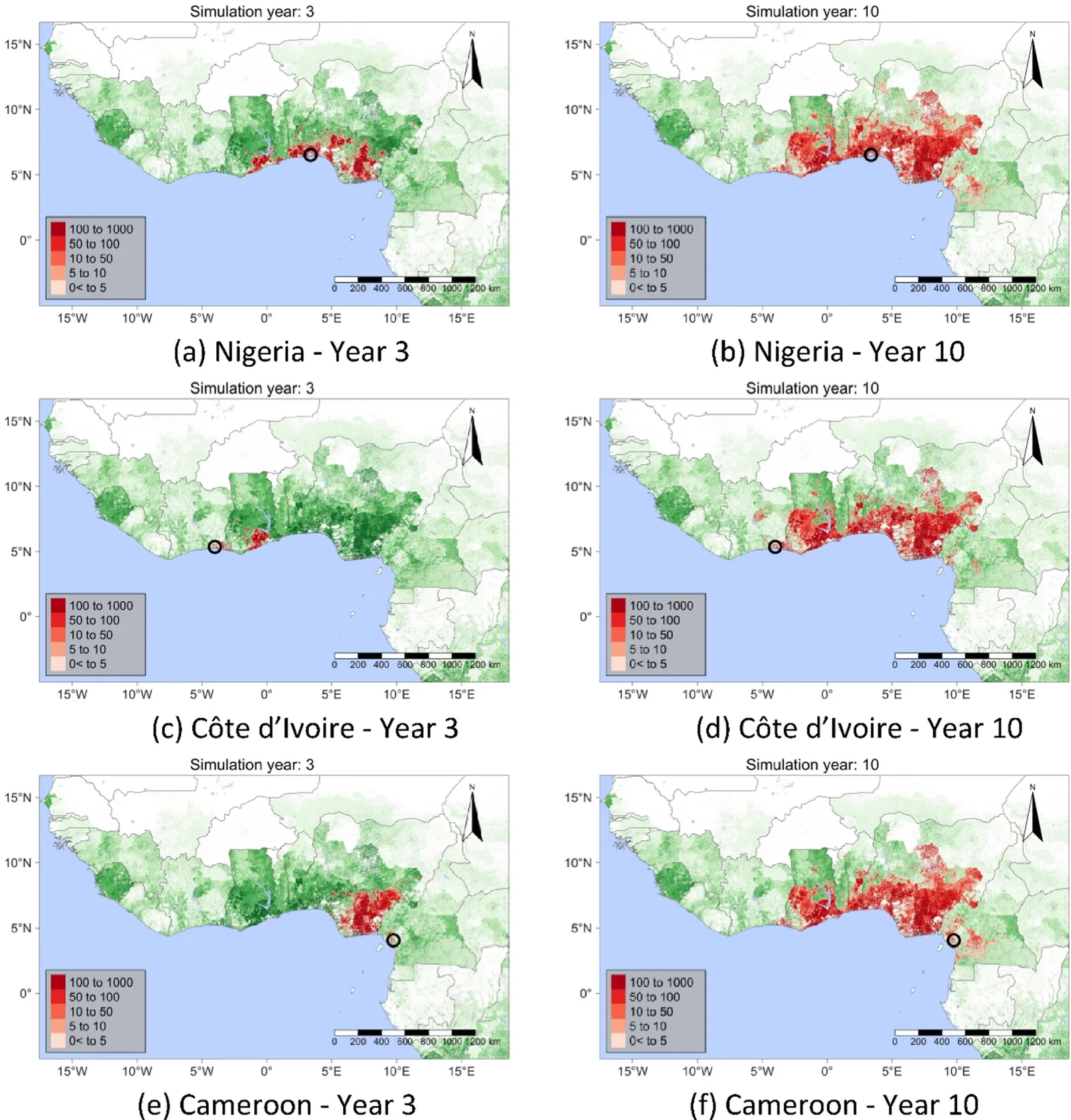
A single realisation of the model showing the median simulation of epidemic spread resulting from the direct introduction of infected cuttings (equivalent of five separate cassava fields – see Methods) to the area surrounding major and well-connected cities in West Africa. Black circles indicate the area in which initial infected fields were located. Maps were produced in R 4.2 (https://www.R-project.org/) using the tmap v4.0 package (https://doi.org/10.18637/jss.v084.i06).

In the Nigeria scenario, simulations show rapid spread across southern Nigeria and into Benin and Ghana by year 3, with over 10% of cassava fields affected, reflecting high cassava densities in these countries, with lower levels of infection in Togo and Cameroon where cassava density and connectivity are lower (Fig. 2a). After 10 years, the epidemic is predicted to have bulked up rapidly in the three countries (Nigeria, Benin and Ghana) that experienced rapid spread within 3 years but to have not greatly expanded beyond this region (Fig. 2b).

**Fig. 3:**
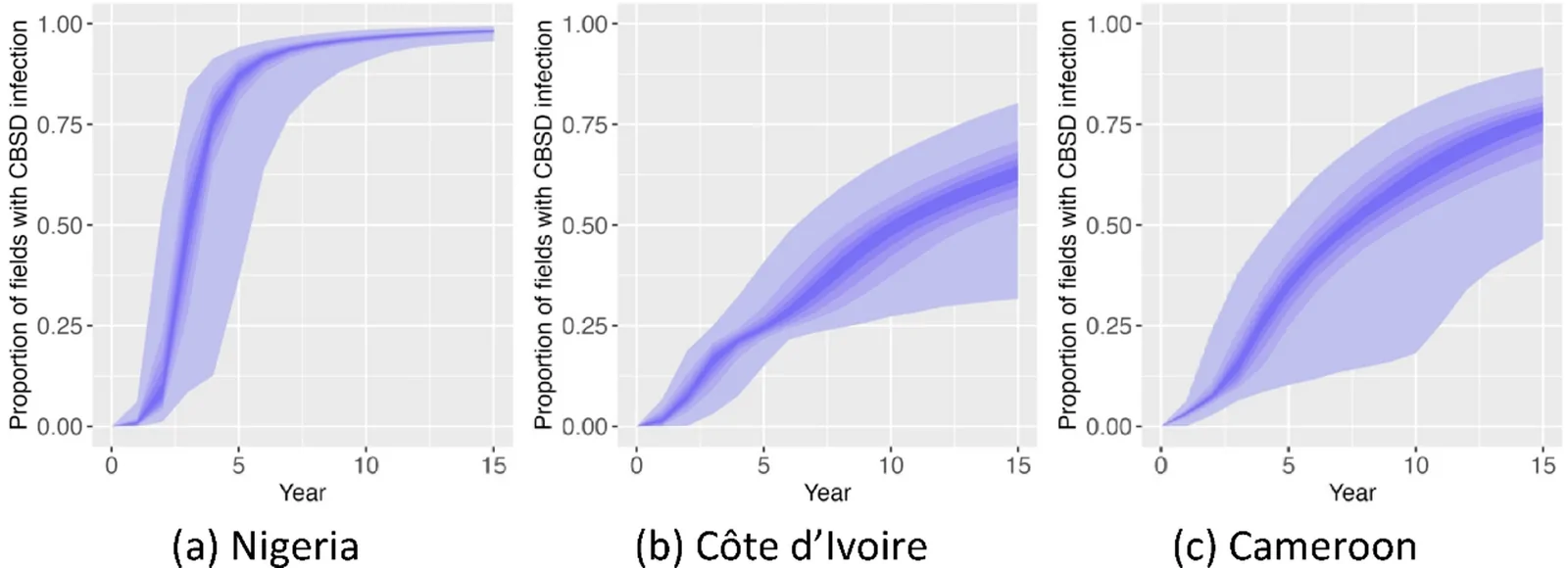
Disease progress curves (DPCs) from approximately 1000 direct introduction simulations wherein the equivalent of five separate infected cassava fields were introduced to the area surrounding major and well-connected cities in West Africa. DPCs restrict calculations of disease bulk up to the respective country of disease introduction. Each gradation in shading indicates ten percentiles of the distribution of infection levels, with the exception of the central band encompassing the 40–60 percentile range.

With epidemic initialisation in Côte d’Ivoire, we saw relatively slow bulk up within Côte d’Ivoire by year 3 but the epidemic rapidly spread in southern Ghana (Fig. 2c). By year 10, the model predictions indicate that the epidemic has spread extensively into countries to the east and the distribution appears almost indistinguishable from the scenario where infection is initialised in Nigeria (Fig. 2d). In the Cameroon scenario, infection bulks up relatively slowly within the country, but rapidly spreads in southeastern Nigeria (Fig. 2e), with a similar regional distribution emerging by year 10 as observed in Nigeria and Côte d’Ivoire scenarios (Fig. 2f). The initial site of direct introduction appears to have little impact on the 10-year spatial distribution of infected fields due to the high density and connectivity of cassava cropping across this region of West Africa (Figs. 2b, d and f and 3), with all introduction sites reaching at least 50% prevalence across West Africa within this timeframe.

### Predicting the present-day distribution and future cross-continental spread of the CBSD epidemic

**Fig. 4:**
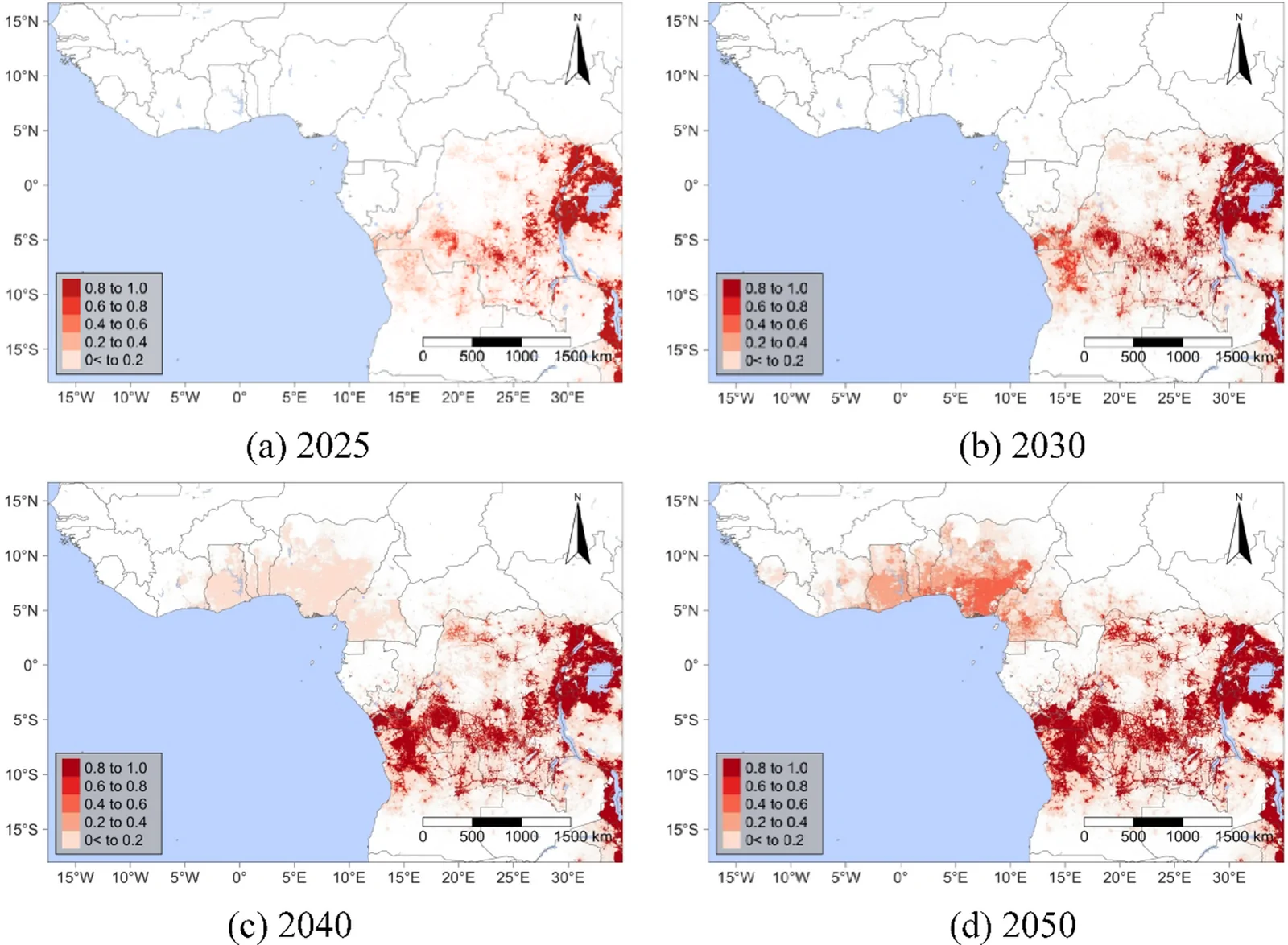
Risk maps representing the probability that any fields within a given one km^2^ raster cell will be infected with CBSD from (**a**): the present Day (2025) through subsequent decades to (**d**): 2050. Maps were produced in R 4.2 (https://www.R-project.org/) using the tmap v4.0 package (https://doi.org/10.18637/jss.v084.i06).

Given the extreme sparsity of CBSD surveillance data relative to the scale of the epidemic, the exact present-day distribution of the epidemic is not known throughout East and Central African countries. We now predict the present-day distribution of the epidemic, and from there move to making predictions of future cross-continental spread over subsequent decades. From a set of 9072 simulations covering the period from 2004 to 2054, we isolated the subset of 414 simulations that correspond to all real-world reports of CBSD spread (Fig. 1a) and bulk up within Uganda^11,13,14,17,18,28^. Each simulation initialised in 2004 that is consistent with survey results to the present day provides a plausible continent-wide infection status, most importantly filling in unsurveyed locations (see Methods for further information). The predicted present day (taken as 2025) CBSD distribution incorporates the historically endemic region of coastal East Africa and Malawi as well as the newly endemic regions of Uganda, western Kenya, north-western Tanzania, Rwanda, Burundi, and Zambia (Fig. 4a). Considering the post-2004 spread west of Uganda up to the present day, our simulation results indicate that the majority of eastern DRC is highly likely (probability above 50%) to be endemic, with a high probability of presence in the region surrounding Kisangani in north-central DRC. Most notably, predictions indicate likely spread further west of current reports, specifically extensive spread across southern DRC (probability above 40%) and into north-eastern Angola (probability above 20%).

**Fig. 5:**
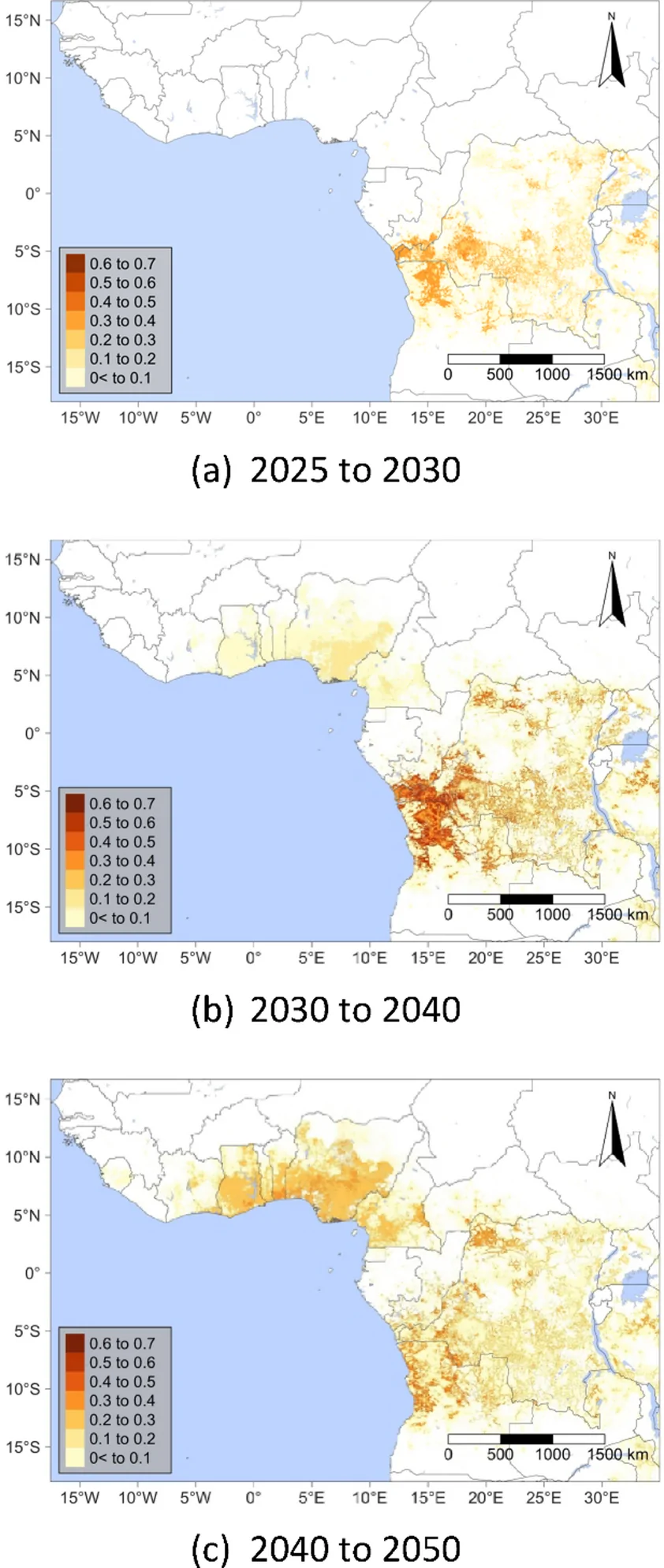
Differential risk maps representing the change in risk probability across the risk maps in Fig. 4, highlighting regions that are likely to experience the most significant increase in the probability of CBSD occurrence across each time period. Maps were produced in R 4.2 (https://www.R-project.org/) using the tmap v4.0 package (https://doi.org/10.18637/jss.v084.i06).

By 2030, risk maps predict extensive CBSI epidemic expansion beyond the present day predicted distribution in northern Angola, south-western DRC, and north-central DRC, representing more than a fivefold increase in expected infected area compared with 2025. Likely new regions include Cabinda in Angola and southern Congo-Brazzaville (Fig. 4b). Figure 5a highlights the major regions of change predicted by the simulations in the form of a differential risk map, which is derived by subtracting the risk map in 2025 from the risk map in 2030.

By 2040, risk maps show expansion in north-western DRC and new incursions in the Central African Republic (CAR) and Gabon. Stochastic model simulations indicate approximately a 15% chance of CBSI having arrived in both Cameroon and Nigeria, with potential spread as far west as Côte d’Ivoire (Figs. 4c and 5b).

By 2050, in the absence of effective control programmes, the model predicts extensive epidemic spread in West Africa, with 45% of simulations reaching Nigeria (Figs. 4d and 5c). In addition, Supplementary Movie 1 illustrates a representative single realisation of the spatiotemporal dynamics of CBSD spread across the continent.

### Analysing the variability of predicted spread rates and arrival times

**Fig. 6:**
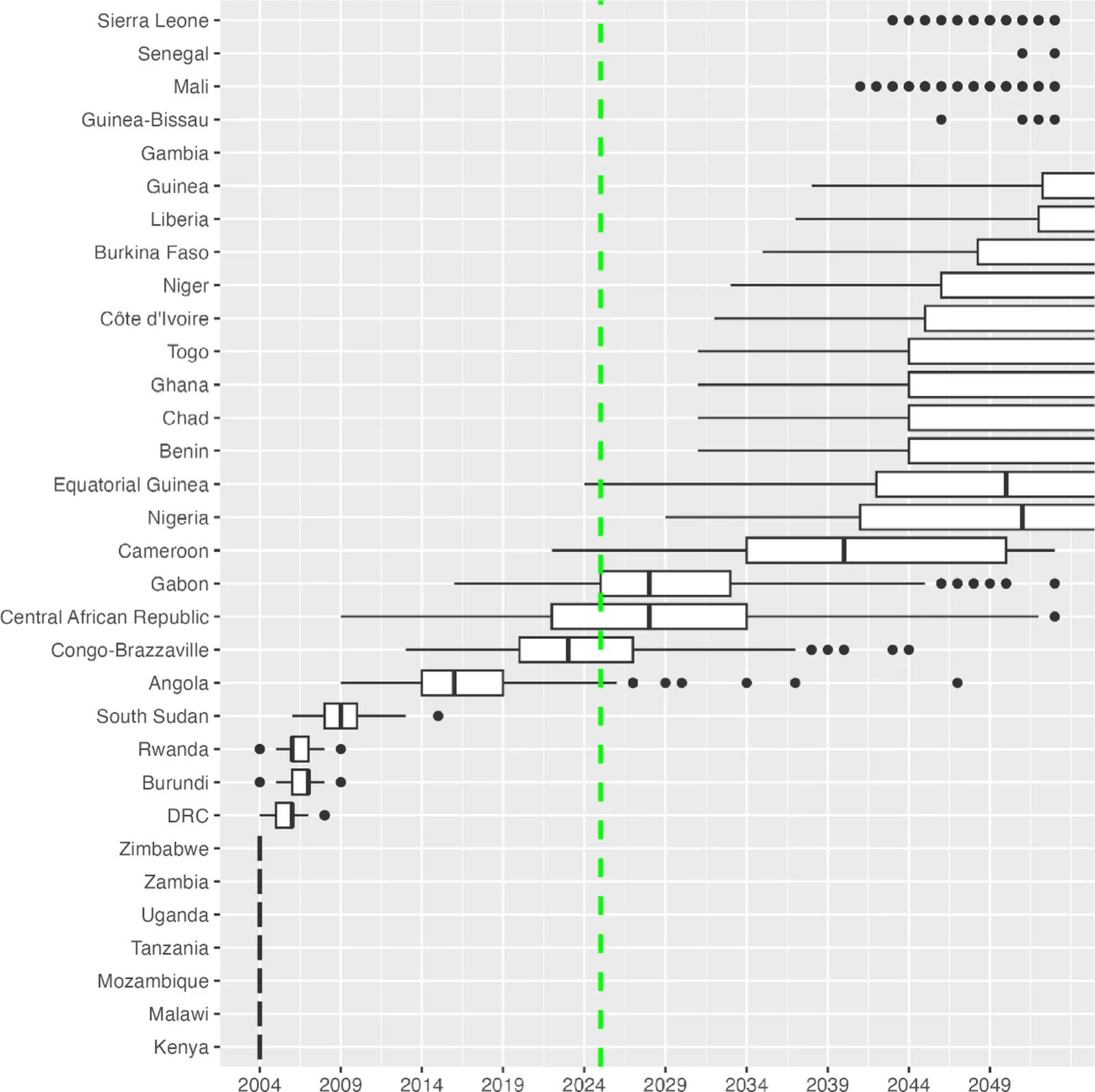
Box plot summarising the predicted year of CBSD arrival in the cassava growing countries of sub-Saharan Africa. The green line indicates the present Day (2025). The central line corresponds to the median and the hinges correspond to the 25th and 75th percentiles. The whiskers extend to the largest and smallest values that fall within the 1.5 x IQR, where IQR is the inter-quartile range between the 25th and 75th percentiles. Points outside this range are plotted as outliers.

The predicted arrival times of CBSI in each of thirty-two major cassava growing countries in SSA are summarised in Fig. 6. The arrival times are based upon simulations initialised in the endemic regions that also correspond to the Uganda surveillance data from 2004 and reported spread beyond Uganda (see Methods). The variability in predictions reflects the intrinsic stochasticity of the epidemic model and the uncertainty in the estimated parameters. There is minimal variability for CBSI arrival times in countries neighbouring Uganda, with the uncertainty generally increasing for more distant countries.

**Fig. 7:**
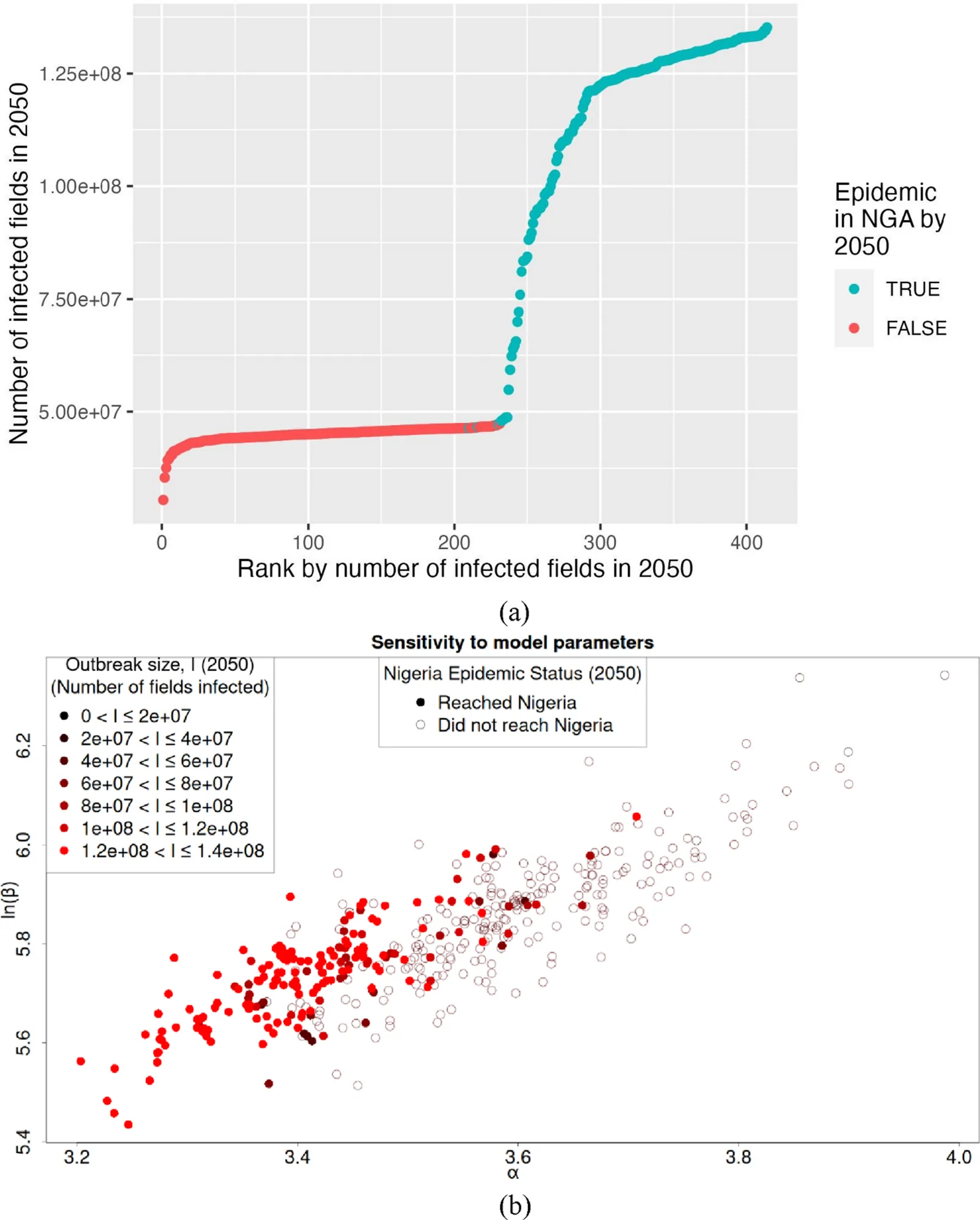
(**a**) The predicted size of the epidemic in 2050 in terms of the total number of fields in sub-Saharan Africa infected with CBSD. Each point represents a single simulation, coloured according to whether there are any CBSD infected fields in Nigeria by 2050. There are two distinct predicted scenarios in terms of the size of the resultant epidemic, with Cameroon acting as a major gateway from Central into West Africa and Nigeria as a major booster in the rate and extent of epidemic spread. (**b**) Effect of model parameters on predicted spread by 2050. The points represent samples of the (log) spread rate (ln(β)) and the dispersal kernel power (α) from the fitted posterior distribution. Filled circles indicate simulations in which the epidemic reaches Nigeria by 2050, and the shading of points indicates the severity of the outbreak, ranging from black for no outbreak to red being the most severe. The simulations in which the epidemic reaches Nigeria are predominantly associated with parameters with low α, corresponding to a longer scale for individual dispersal events.

There is a step change in the total size of epidemics that have reached Nigeria (Fig. 7a), with Cameroon acting as a major gateway from Central into West Africa and Nigeria as a major booster in the rate and extent of epidemic spread. Supplementary Movie 2 ranks simulations by epidemic size (number of fields infected in 2050) and illustrates the relatively broad spatial variability in predicted spread. Simulations showing the most extensive westward spread tend to have lower values of the dispersal kernel scale parameter α, corresponding to longer average dispersal distances (Fig. 7b). Due to the correlation between α and ln(β) (log of the spread rate) from the fitting process, low values of α are necessarily associated with low values of ln(β); the extent of westward spread is therefore primarily determined by α. Figure 7b shows the clustering of simulation parameters for which epidemics reached Nigeria at lower values of α (i.e. longer average dispersal distances) (See Supplementary Information or the original model publication^12^ for a full explanation of the modelparameters).

## Discussion

Since 2004, CBSD has spread from Uganda over 1000 km to Zambia in the south and to central DRC in the west, causing significant loss of yield for smallholder farmers in the region^10^. The westward spread of CBSD poses a serious threat to food security in countries reliant on cassava as a staple crop. To address this, we adapted and extended a stochastic epidemiological model, previously validated for spread across Uganda, to predict the present extent and future spread of CBSD across all thirty-two major cassava growing countries in SSA. The predictive accuracy of the extended model was validated relative to real-world reports of CBSD spread in East, Central and Southern Africa up to the present day, with the model showing strong correspondence to the timing of real-world observations (Fig. 1b). These data on real-world observations of the disease beyond Uganda were then incorporated into the analysis to improve predictive accuracy and reduce the uncertainty in model predictions (Fig. 1c).

The predicted cross-continental epidemic spread rates are the result of the relatively local (i.e. < 50 km) combined action of the two CBSI dispersal mechanisms: the insect vector, *B. tabaci* and human-mediated movement of infected cuttings. The epidemic spread observed in Uganda and regionally in East Africa is consistent with the combined action of these two, relatively local, dispersal mechanisms. However, due to the long-term viability and persistence of the virus in cuttings, there is a risk of much longer-range introduction of CBSI to disease-free regions via cutting movement. Termed direct introduction, these movements can occur by air, sea or long-range road transport. To illustrate the scale of this risk for West Africa, we applied the model to direct introduction scenarios wherein the equivalent of five infected fields were introduced near each of the three major ports in Nigeria, Côte d’Ivoire, and Cameroon. Spread rates vary significantly by country, driven by host density, landscape connectivity, and vector abundance. Consistent with this, infection initially spreads more slowly from direct introductions in Côte d’Ivoire and Cameroon than Nigeria (Fig. 3). Despite the varying local cassava densities and *B. tabaci* abundance leading to differences in spread rates, these three scenarios all indicate that epidemic spread would be far more rapid than seen to date in East Africa and result in extensive infection across West Africa, irrespective of the original source of introduction (Figs. 2 and 3). As such, it is of vital importance to prevent introductions to West Africa. Nonetheless, even in the absence of spread in West Africa, all simulations predict extremely extensive epidemic spread throughout East and Central Africa, impacting millions of smallholders.

While the risk of direct introductions may be low with phytosanitary restrictions at seaports and airports, the potential harm of direct introductions could be extremely severe if the disease were to establish and spread prior to detection and response. Large-scale cutting movements by air for humanitarian purposes have been documented^27^. The risk of unintended consequences of importation of disease along with planting material merits careful assessment. The rapid spread and lack of advance warning from presence in neighbouring countries emphasises the need for proactive and pre-emptive establishment of surveillance and disease management capabilities and merits the consideration of the direct introduction scenario separately to progressive cross continental spread.

The scarcity of reports of CBSD epidemic expansion in East Africa and especially in Central Africa means there is significant uncertainty about the present-day distribution of the pathogen and disease. Therefore, for predictions of the future spread of the epidemic, the model could not be directly initialised on the likely present-day extent of infection as this distribution is not known (see Methods). To estimate the present-day epidemic across East, Central and Southern Africa, we initiated simulations from Uganda in 2004 and retained only those simulated epidemics whose outputs matched the location and timing of the real-world reports beyond Uganda. Given the sparsity of reporting, we could only be confident that CBSI was present at the time of the report, as opposed to assuming CBSI absence prior to the first report. Matching simulations to reported presence allowed us to discard simulations that spread too slowly, but provided no upper bound to exclude those that spread too rapidly. However, we also imposed a constraint whereby selected simulations were also required to conform to surveillance data in Uganda from 2004 to 2017, which effectively introduced both upper and lower bounds on spread rates. By contrasting Fig. 1b with Fig. 1c, we see the improved prediction accuracy for arrival times in Central DRC by incorporating additional information on real-world observations.

Regarding the CBSD endemic region in coastal East Africa and Malawi, we assumed there was no fundamental distinction between the endemic and post-2004 epidemic regions, which is consistent with available data on the CBSI species distribution^14,16,29,30^. Preliminary simulations with and without seeding infection in the endemic coastal East African and Malawi regions showed that the endemic region contributed minimally to the post-2004 epidemic spread. Nonetheless, to gain a complete picture of the spatial extent of CBSI infection in SSA, we included the endemic regions as sources of infection. However, data for the true distribution of infected fields in the endemic region were limited, as was information on the post-2004 epidemic spread, which meant it was also necessary to make several assumptions to arrive at a credible distribution of infected fields in the endemic region (see Methods).

With the harmonised surveillance protocols and consistent surveillance efforts of the co-authors from the CDP and WAVE projects, we have high confidence in a consistent delay between epidemic introduction to a new region and outbreak detection. This gives a robust framework for evaluating the accuracy of model predictions. The range of outcomes of the stochastic model allows for the exploration and quantification of the likelihood of particular outcomes given the uncertainties within the available data.

In the absence of contrary evidence, we assumed that there is nothing fundamentally distinct about cassava cultivars or *B. tabaci* diversity in West Africa that would prohibit the spread of CBSI. Studies on the susceptibility of West African cassava varieties have demonstrated susceptibility to CBSI^22,23^ and to the authors’ knowledge there are no published studies that show any significant populations of CBSI-resistant cassava varieties. Further studies are needed to characterise the response of cassava cultivars to CBSIs and to map the spatial distribution of cassava cultivars. Once available, this information could be incorporated into the model to improve local prediction accuracy. Future work is underway to investigate optimising the deployment of clean and resistant planting material. Although *B. tabaci* exhibits species level diversity across SSA^31^, we assume equivalent transmission efficiency across all species within the complex, with variation in spread rates driven by vector abundance rather than vector species. To the authors’ knowledge, no published studies have quantified CBSI transmission efficiencies across the *B. tabaci* complex. Were this information available it could be incorporated in future models to modulate local spread rates accordingly, with implications also for modelling of disease management interventions.

With major uncertainties in climate change, demographics, and agricultural trends, we do not know how cassava production will change in the future. As a result, the model currently assumes a fixed level of cassava production across time. Future studies could incorporate changes in cassava cultivation associated with human population growth, though predicting the spatial distribution of both population growth and cassava cultivation under climate change remains a significant challenge^32^. The choice to use CassavaMap^33^ rather than MapSPAM^34^ was motivated by the need for a finely resolved and accurate host map, suitable for resolving targeted disease management interventions. The 10 km resolution of the MapSPAM dataset, along with lower accuracy in host presence and density^35^, motivated the use of the 1 km resolution CassavaMap dataset.

Despite incorporating data from over 18,000 survey observations, the *B. tabaci* abundance records are sparse at the continental scale, especially in Central Africa. Future work can mitigate the challenge of limited vector data by modelling the *B. tabaci* abundance explicitly. Further work is underway to improve the inverse distance weighting (IDW) interpolation used to generate the vector abundance layer used here (see Methods). This work will link weather data with a whitefly population dynamics model to generate dynamic, environmentally driven predictions of vector abundance for use in future epidemic models. These meteorologically driven models can also be linked to future climate scenarios (IPCC RCPs) to explore how projected climate change may affect vector abundance. Moreover, the CBSI epidemic spread model currently integrates the two dispersal processes (i.e. vector and human-mediated cutting movement) into a single dispersal kernel due to the absence of data to parameterise these two processes independently^12^. The lack of detailed data on trade networks for cassava planting material movement^26^ and the importance of both the trade and whitefly dispersal components for significant outbreaks^36^ is an area where future studies should attempt to collect sufficient data on cutting trade movements to begin to disentangle these two mechanisms. While we demonstrate here that a blended dispersal kernel is sufficient for predictions of epidemic spread, separate information on trade movements would allow increased precision in future work for modelling of epidemic management interventions. The Eastern and Central African regions at the centre of much of the epidemic spread have been subject to significant political instability and large-scale movements of human populations. Incorporating these factors into future models as data on human-mediated movements of planting material become available could lead to improved inferences for mechanisms of epidemic spread.

The mechanistic structure of the underlying epidemiological model used in the current analyses is well suited to compare scenarios for early detection and response. Phytosanitary treatments, such as quarantine, roguing, culling and the deployment of virus-free planting material as well as insect vector control can be easily simulated within the modelling framework and the likely dynamical effectiveness of different scenarios compared.

Our current predictions provide an important starting point in mobilising resources to prepare for and mitigate spread. The predictions are necessarily subject to uncertainties. On longer timescales there are likely to be significant changes to host, vector, climate and society, each of which could affect epidemic spread. The current model based upon a static cassava map is likely to predict slower spread rates compared with a future landscape with increased cassava cultivation density. Therefore, it is expected that likely future trends in the drivers of the epidemic would increase spread rates on the longer timescales. Subject to these uncertainties, the model has demonstrated that it is possible for CBSD to spread across the continent, establish and rapidly multiply in West Africa, under current conditions, and so the slowing or prevention of epidemic spread will require a significant change to the status quo.

The most pressing question facing countries in disease-free regions is how to prepare for the threat of CBSD introduction in a way that makes the most efficient use of limited resources. The model described in this study not only provides estimates for arrival times acrossSSA but lays the foundations for a continental-scale quantitative framework wherein both surveillance and management options can be explored and optimised. The results presented here highlight three priorities: strong phytosanitary restrictions to prevent the direct introduction of infected planting material to West Africa, effective surveillance for early detection of CBSI incursions and upon detection rapid responses at appropriate scales.

## Methods

### Extending the data-driven layers of the CBSD model

We took a parameterised model of the CBSD epidemic in Uganda^12^ and extended the data-driven input layers using additional data on cassava host production and *B. tabaci* abundance from across SSA. For the host landscape layer, we used the CassavaMap model^33^, which covered the 32 major cassava producing countries in SSA and converted from production volume per km^2^to number of fields per km2 NaN^33^,. When referring to a cassava field, we used a representative unit field size of 0.1 ha, yielding approximately one tonne of cassava root material. Cassava was assumed to be of uniform susceptibility across SSA^22,23^, though this remains an area of active scientific investigation.

For the *B. tabaci* vector abundance data, we combined 18,340 vector abundance records from cassava disease surveys in 17 countries across SSA, reporting the average field-level abundance of *B. tabaci.* The vector abundance data were partially compiled from previous publications^11,17,37–51^(see Table 1 for details), with additional unpublished data provided by co-authors. These data were collected using the same in-field protocol as the Ugandan surveillance data and the relative vector abundance layer was generated using inverse distance weighted (IDW) interpolation with a power value of 1.0 at a 5 km resolution at the same spatial extent as the host production layer^12^.

**Table 1:** Summary of the number of field records used to generate the *B. tabaci* abundance model layer. These data were partially compiled from multiple publications along with previously unpublished data provided by co-authors. A subset of these data was also used to seed locations for CBSD in the endemic region of coastal East Africa and Malawi.

Country	Number of field surveys	Date range of field surveys used in whitefly layer	Relevant citations
Benin	232	2015–2022	(Houngue et al., 2022)^49^
Burkina Faso	276	2015–2022	(Soro et al., 2021)^48^
Cameroon	342	2020–2022	(Doungous et al., 2022)^50^
Côte d’Ivoire	853	2016–2022	
DRC	1326	2009–2022	(Zeyimo et al., 2019; Muhindo et al., 2020b)^17,46^
Gabon	227	2020–2023	(Mouketou et al., 2022)^51^
Ghana	701	2015–2022	(Oppong et al., 2020)^47^
Kenya	505	2009–2017	(Ateka et al., 2018)^40^
Malawi	296	2012–2015	(Mbewe et al., 2018)^44^
Mozambique	1226	2012–2017	(Cossa et al., 2018)^42^
Nigeria	2127	2015–2022	(Eni et al., 2018, 2020, 2021)^37–39^
Rwanda	348	2009–2017	(Kanyange et al., 2018)^43^
Sierra Leone	268	2020–2022	
Tanzania	809	2009–2017	(Tairo et al., 2018)^40^
Togo	344	2015–2020	
Uganda	7507	2004–2017	(Alicai et al., 2019)^11^
Zambia	953	2009–2019	(Chikoti et al., 2018)^41^

### Initial conditions for the epidemic state in 2004

For all model simulations of cross continental spread, we defined the start time as 1 st January 2004, which we took to be the start of the Ugandan CBSD epidemic. To generate a model layer representing the distribution of CBSD-infected fields across all cassava-producing countries in 2004, it was necessary to account for the pre-2004 CBSD endemic region. Cassava brown streak disease has been reported as endemic in coastal East Africa and the shores of Lake Malawi since the first scientific reports beginning in the 1930s^52–54^. For the coastal countries of Kenya, Tanzania and Mozambique, polygons were defined covering a 2 decimal degree wide region inland from the coast (approximately 220 km). In the case of Malawi, due to its smaller size, the entire country polygon was used. These polygons were overlaid with the surveillance dataset (described in the previous section) to identify CBSD-positive fields observed within the endemic region (Supplementary Fig. 1a).

To create a layer representing the distribution of infected fields in the endemic region, we ran preliminary simulations to produce a plausible spatial distribution of infection beyond the locations captured in the surveillance data. For the initial conditions in these preliminary simulations, the equivalent of a single infected field was seeded at the location of each of the CBSD positive surveys from the surveillance data. An ensemble of 100 preliminary simulations was run using these initial conditions for 20 years. Field surveys in the endemic polygons were approximately 50% CBSD positive. Therefore, we discarded simulations in which less than 50% of fields within each endemic polygon were infected, as well as those that exceeded 1% of infected fields in any non-endemic country. From the subset of simulations that met this criterion, a simulation was selected at random, and the 20-year end state was taken as representative of the 2004 CBSD distribution in the endemic region (Supplementary Fig. 1b).

Initial conditions for the 2004 state were derived by combining the endemic region infection layer with the initial CBSD observations in Uganda near Kampala in 2004^11^.

### Running cross-continental simulations from 2004 to 2054

Taking the initial conditions representing the epidemic state in 2004, we ran an ensemble of 10,000 simulations for a 50-year period using the University of Cambridge HPC service. Due to the computational demands of these simulations, we applied a one-month time-to-completion cut-off. Of the 10,000 simulations, 9,072 completed within this time limit.

### Initial conditions for cross-continental predictions beyond the present day

We used the observations of CBSD spread within and beyond Uganda to isolate the subset of stochastic simulations that corresponded to the real-world observations up to the present day (Fig. 1a). In doing so, we constrained the predictions of epidemic spread beyond the present day to be consistent with available information about the state of the system up to the present day. We adopted this approach for two reasons: the true present-day initial conditions are unknown due to sparse real-world observations, and extrapolating from surveillance data to the true underlying state is inherently uncertain.

We utilised the simulated surveillance features of the model to simulate CBSD surveillance at the same time and locations as the real-world Ugandan surveillance^11^. We then calculated two summary statistics: the proportion of positive survey results within 50 km of Kampala, *S*_*cen*_, and the equivalent proportion nationally across Uganda, *S*_*nat*_, collectively referred to as *S*_*inf*_ across the full 2004–2017 dataset period. Simulations that satisfied a tolerance of ε_*inf*_ on both statistics were retained^12^.

For the set of observations beyond Uganda (Fig. 1a), we identified simulations that had any CBSD infected fields within a polygon corresponding to the area of the real-world CBSD observations by the end of the year of real-world observation. For the two reports from single sites with a specified latitude-longitude coordinate, labelled DRC (East) and DRC (Central) in Fig. 1a, circular polygons were created with a radius of 100 km around the specified coordinate; for Rwanda and Burundi, the entire country polygons were used; for DRC (southeast), a polygon of Pweto territory was used; and for Zambia, we created a single polygon from the union of Chiengi and Kaputa districts.

Analyses were then performed on the subset of simulations that met all specified requirements (i.e. corresponded to Ugandan surveillance data and reported spread beyond Uganda). Out of the 9,072 simulations, a total of 414 met all these criteria.

### Direct introduction predictions

Independent direct introduction simulations were seeded for each specified starting location with five independent infected field sites, matching the scale of the initial CBSD outbreak detected in Uganda in 2004. The starting date was not specified to allow interpretation as if introduced at any point within the decade following 2025. Simulations were run for 15 years post introduction.

## Supplementary Information

Below is the link to the electronic supplementary material.

Supplementary Material 1

Supplementary Material 2

Supplementary Material 3

## Data Availability

The field record datasets collected in East Africa as part of the Cassava Diagnostics Project (CDP) are made available as part of the code repository. The remaining field datasets provided by the Central and West Africa by the Central and West African Virus Epidemiology Center (WAVE) are available upon reasonable request from WAVE.

## Code availability

The R code used for analysis and figure generation in this paper and usage instructions are available at https://github.com/dsg38/cbsd_scenarios/ The C++ code for the simulations is freely available as described in ^**12**^.
