# Supplementary material for: Predicting the cross-continental spread of the cassava brown streak disease epidemic in sub-Saharan Africa
Source: Sci Rep. 2026 Jul 2;16:30402. doi: 10.1038/s41598-026-59438-2 (PMC13630549; doi:10.1038/s41598-026-59438-2)
Supplement: Supplementary file 1 [file 41598_2026_59438_MOESM3_ESM.docx]

Supplementary information

**Predicting the cross-continental spread of the cassava brown streak disease epidemic in sub-Saharan Africa**

David Godding^1†*^, Richard O. J. H. Stutt^1†**^, Merveille Koissi Savi^2^, Corneille Ahanhanzo^2^, Fidèle Tiendrebeogo^2^, Oumar Doungous^3^, Monde Godefroid^2^, Zeyimo Bakelana^2^, Jacques François Mavoungou^2,4^, Allen Oppong^2,5^, Elijah Ateka^6,7^, Willard Mbewe^8^, Nurbibi Cossa^9^, Chukwuemeka Nkere^2,10^, Ibrahim Umar Mohammed^2,11^, Marie Claire Kanyange^12^, Alusaine Edward Samura^2,13^, Adjata Djodji^2^, Titus Alicai^14^, Patrick Chiza Chikoti^15^, Fred Tairo^16^, Peter Sseruwagi^16^, Joseph Ndunguru^16^, Angela Obiageli Eni^2^, Justin Simon Pita^2^ and Christopher A. Gilligan^1^

^1^Epidemiology and Modelling Group, Department of Plant Sciences, University of Cambridge, Cambridge, CB2 3EA, UK

^2^Central and West African Virus Epidemiology (WAVE), Scientific and Innovation Hub of Bingerville, Félix Houphouët-Boigny University (UFHB), Abidjan 22 BP 582, Côte d’Ivoire

^3^Central and West African Virus Epidemiology (WAVE), Biotechnology Laboratory, Ekona Regional Research Centre, Institute of Agricultural Research for Development, PMB 25, Buea, Cameroon

^4^Institut de Recherches Agronomiques et Forestières (IRAF), B.P : 2246, Libreville-Gabon

^5^CSIR Crops Research Institute, P. O. Box 3785 Kumasi, Ghana

^6^Department of Horticulture, Jomo Kenyatta University of Agriculture and Technology, Nairobi, Kenya

^7^Jaramogi Oginga Odinga University of Science and Technology, Bondo, Kenya

^8^Department of Biological Sciences, Malawi University of Science and Technology, P. O. Box 5196, Limbe, Malawi

^9^Mozambique Agricultural Research Institute, Maputo, 2698, Mozambique

^10^National Root Crops Research Institute (NRCRI) Umudike, Km 8 Ikot Ekpene Road, PMB 7006 Umuahia, Nigeria

^11^Abdullahi Fodio University of Science and Technology, Aliero (AFUSTA), Along Sokoto Road, Aliero, PMB 1144 Kebbi State, Nigeria

^12^Rwanda Agriculture Board, PO Box 5016 Kigali, Rwanda

^13^Department of Crop Protection, School of Agriculture and Food Sciences, Njala University, Njala Campus, Njala, Sierra Leone

^14^National Crops Resources Research Institute, Namulonge, Kampala, Uganda

^15^Zambia Agriculture Research Institute, Ministry of Agriculture, Chilanga, Zambia

^16^Mikocheni Agricultural Research Institute, P.O. Box 6226, Dar es Salaam, Tanzania

^†^These authors contributed equally to this work.

^*^email: david.godding@outbreaklabs.co

**email: rs481@cam.ac.uk

# S1 Supplementary Methods

## Endemic regions pre-2004

Regions with endemic infection of CBSI prior to the post-2004 CBSD epidemic in Uganda are recorded in Figure S1.

(a)	(b)

Supplementary Figure 1: (a): An overview of the regions that are recorded as endemic for CBSD prior to the post-2004 CBSD epidemic in Uganda. Black points represent the locations of CBSD-positive field surveys used to seed infection to generate a realistic layer of infection for the 2004 state of CBSD infection in the endemic region. (b): The initial conditions taken to represent the distribution of CBSD infected fields at the start of the 2004 Ugandan CBSD epidemic. Red gradient indicates the proportion of cassava fields in a given 1 km^2^ raster cell that are infected with CBSD. Red cross indicates the site of the initial 6 fields recorded as CBSD positive in Uganda. Maps were produced in R 4.2 (https://www.R-project.org/) using the tmap v4.0 package (https://doi.org/10.18637/jss.v084.i06).

## Model structure

The epidemiological model used has been described fully in Godding et al. (2023). Summarised here for convenience, the model used is a stochastic metapopulation epidemic model for CBSD at a 1 km^2^ resolution. The model integrates a host landscape of cassava production (Szyniszewska, 2020) and the relative spatial abundance of the insect vector, B. tabaci at a 1 km^2^ resolution, obtained from interpolation of survey data. Spread of CBSD across the host landscape is a raster-based spatially explicit, SI (Susceptible-Infected) epidemic. An SI compartmental structure was selected as cassava is a vegetatively propagated crop, so infection persists from one harvest to the next planting. The model was parameterised and validated using annual surveillance data for the spread of CBSD in Uganda (Alicai *et al.*, 2019).

The instantaneous state of the model is defined by the number of susceptible and infectious fields in each raster cell. The state is updated via a discrete event, continuous time stochastic process using an optimised Gillespie algorithm. Spatial coupling between infected and susceptible cells is governed by an isotropic discrete power law dispersal kernel, *K*, where *d_ij_* is the distance in km between the centroids of two raster cells *i* and *j* and α is the exponent and so:

$$K\left( d_{ij} \right)=Ad_{ij}^{-\alpha}$$

Due to an absence of data independently quantifying the two dispersal mechanisms, the model integrates both the net effects of dispersal by *B. tabaci* and the movement of planting material on the spread of infection into the single kernel. An additional parameter, *p*, defines the kernel value at *d* = 0, where *p* is the proportion of dispersed inoculum that remains within the source cell. A kernel cut-off distance, *D*_max_ = 500 km, sets the maximum distance from the source cell that the kernel covers. For the finite set of cell centroids in the range 0 < *d* ≤ *D*_max_, values are calculated based on the kernel function. A normalisation factor, *A*, is applied such that the sum of kernel values for *d* > 0 equals 1 − *p*. Therefore, the sum of the kernel over all cells within a distance *D*_max_ is 1.

The force of infection at location *i*, $\varphi_{i}$, incorporates the kernel function*, K*, the transmission rate, β, the vector abundance parameter at location *j*, *w_j_*, and the current number of hosts in the infectious state, *I*_j_, where *j* represents all locations in the rasterised landscape, including *i*.

$$Force of infection at location i: \varphi_{i}=\sum_{j} \beta w_{j}I_{j}K(d_{ij})$$

The instantaneous rate of infection at a given raster cell, *i*, from all locations, *j*, is $\psi_{i}$, that incorporates the vector abundance parameter, w_i_, and the number of susceptible hosts, *S_i_*, at location *i*.

$$Infection rate at location i: \psi_{i}=\varphi_{i}w_{i}S_{i}$$

The effect of an infection event at location *i* is given by

$$Effect of infection event at location i: S_{i}\to S_{i}-1, I_{i}\to I_{i}+1$$

We assume a linear relationship between vector abundance and its effect on infection and field-level susceptibility up to a field-level mean of 20 *B. tabaci* per plant.

Key model assumptions: The model simulates only one type of host, vector and virus. Hence, we assume that all cassava fields in the model are equivalently susceptible to CBSIs and, if infected, have the same probability of being detected if surveyed. We therefore implicitly assume that all species in the *B. tabaci* complex vector CBSIs with equivalent efficiency. Similarly, we implicitly assume that all CBSIs are equivalently infectious. Epidemiological parameter values used in simulations are sampled from posterior distributions derived in Godding et al. (2023) and hence vary amongst simulations but remain constant over time within each simulation. We simulate dispersal using a single dispersal kernel that represents the net effect of both vector and human-mediated forms of dispersal. Whilst both the host landscape and vector abundance layer vary spatially, we assume that both are static in time.

## Supplementary Movie 1

Maps were produced in R 4.2 (https://www.R-project.org/) using the tmap v4.0 package (<https://doi.org/10.18637/jss.v084.i06>) for individual frames of the movie and then combined into an animation using ffmpeg v7.1 (https://www.ffmpeg.org/).

# References

Alicai T, Szyniszewska AM, Omongo CA *et al.*, 2019. Expansion of the cassava brown streak pandemic in Uganda revealed by annual field survey data for 2004 to 2017. *Scientific Data* **6**, 1–8.

Godding D, Stutt ROJH, Alicai T, Abidrabo P, Okao-Okuja G, Gilligan CA, 2023. Developing a predictive model for an emerging epidemic on cassava in sub-Saharan Africa. *Scientific Reports* **13**, 12603.

Szyniszewska AM, 2020. CassavaMap, a fine-resolution disaggregation of cassava production and harvested area in Africa in 2014. *Scientific Data* **7**, 1–5.
